# An integrative review of the impact of mobile technologies used by healthcare professionals to support education and practice

**DOI:** 10.1002/nop2.37

**Published:** 2015-11-27

**Authors:** Ping Guo, Kim Watts, Heather Wharrad

**Affiliations:** ^1^ Cicely Saunders InstituteDepartment of Palliative Care, Policy and RehabilitationKing's College LondonUK; ^2^ Faculty of Medicine and Health SciencesUniversity of NottinghamNottinghamUK

**Keywords:** Clinical practice, healthcare education, healthcare professionals, integrative review, mobile technologies

## Abstract

**Aim:**

The aim of this study was to provide evidence of the impact of mobile technologies among healthcare professionals in education and practice settings.

**Design:**

Integrative literature review.

**Methods:**

Electronic databases including MEDLINE, CINAHL, PsycINFO, EMBASE, ERIC and Web of Science were searched for papers published between 2002–2012. Quantitative studies were critically evaluated based on Thomas *et al*.'s framework, while the consolidated criteria for reporting qualitative research was used to appraise the rigour of the qualitative studies.

**Results:**

Seventeen quantitative and three qualitative studies were included. The findings suggest a largely positive influence of mobile technologies on various clinical practice and educational outcomes. However, robust evidence was limited. Use of mobile technologies in health care are associated with improvements in access to information, accuracy and efficiency, evidence‐based decision making at the point of care and enhancement in performance, confidence and engagement in different contexts.

## Introduction

Since the introduction of mobile technologies in the early 1990s, it has become a valuable and important tool to be incorporated into various medical‐related fields used by multiple disciplines (Ranson *et al*. 2007). Mobile devices can now store large quantities of information. Their operating systems allow applications that support sophisticated user interactions, their graphics capabilities offer representational versatility and their networked status means that they afford easy communication among their users (Walton *et al*. 2005). Moreover, many healthcare professionals (HCPs) will have acquired a degree of familiarity and confidence with such devices through their own personal and recreational uses, so it is easy to argue that this use should be extended to support the education and practice of HCPs.

Various scoping studies (Khan *et al*. 2007, Crook *et al*. 2012, Franko & Tirrell 2012) have identified a range of medical software applications available to HCPs. Most applications are information reference points or quizzes, exam or test your knowledge type software applications. However, evidence of their impact on learning and practice among healthcare professionals is limited.

Several reviews showed that personal digital assistants (PDAs) were being increasingly integrated into clinical practice and medical education (Garritty & El Emam 2006). A PDA is a generic term for any small mobile handheld device including smartphones and tablets that provides computing and information storage and retrieval capabilities for personal or business use, often for keeping schedule calendars and address book information handy (Luanrattana *et al*. 2007). However, none of these provided a comprehensive overview of the use of mobile technologies among healthcare professionals with a focus on its use in improving education and clinical practice. Traditionally, systematic reviews have heavily relied on evidence obtained from quantitative studies. However, this is becoming increasingly important to recognize the benefit of integrating qualitative and quantitative research evidence (Centre for Review and Dissemination 2008). A review can be enhanced when qualitative studies which explore people's experiences and perceptions of a subject area are included. It is particularly true when limited evidence derived from trials is anticipated such as evidence in relation to the impact of mobile technologies. Therefore, to provide a wider overview of this topic, an integrative review was conducted by integrating evidence from both quantitative and qualitative research.

This present review aims to raise the awareness among differing healthcare professionals about the importance of mobile technologies and the potential roles in education and clinical practice settings and provide evidence to support its use. In addition, this review would encourage the further evaluation of the use of mobile technologies and inform the development of specific mobile applications for future education and practice across a range of healthcare professionals.

## Aims

The overarching aim of this review was to identify evidence focusing on the use of mobile technologies in education and practice settings by healthcare professionals. Specifically, the review sought to:

- Examine the impact of mobile technologies on clinical and educational outcomes in healthcare professionals.
- Identify the extent to which quantitative outcomes addressing aspects of clinical and/or educational significance have been used in previous studies.
- Identify the extent to which qualitative evaluation of aspects of clinical and/or educational significance has been explored in previous studies.

Given the aim to capture a comprehensive review of studies in this area, the research question which guided this review was what international evidence exists on the impact of mobile technologies and experience of using mobile technologies in any education and practice settings among different groups of healthcare professionals.

## Methods

### Design

An integrative literature review was conducted to capture all studies evaluating mobile technologies used by healthcare professionals in education and practice settings. Due to the complex nature of this topic, we included all relevant quantitative and qualitative evidence. Review methods recommended by the Centre for Review and Dissemination were adopted to help specify clear and reproducible eligibility criteria for selection of studies, comprehensively search and retrieve relevant studies that met the eligibility criteria, critically appraise the quality of included studies and synthesize findings (Centre for Reviews and Dissemination 2008).

### Search strategy

A comprehensive search strategy was developed to obtain all relevant studies using terms addressing the study focus (e.g. ‘impact’/‘mobile technologies’, ‘smartphones and software applications’, ‘handheld computers’, ‘personal digital assistants (PDAs)’/‘experience’/‘health professionals’). The search was conducted between April and August 2012. The search terms (subject headings or key words) including quantitative (‘evaluation’) and qualitative outcomes (‘perceptions’) were used to map to the title, abstract and full text for identifying both types of studies. Electronic databases including MEDLINE, CINAHL, PsycINFO, EMBASE, ERIC and Web of Science were searched. All searches were screened and duplicated studies were discarded. Reference lists of all retrieved articles were followed up to identify additional studies pertinent to the topic area.

#### Inclusion and exclusion criteria

Studies were included in the review if they met the following criteria: the studies: (1) were published in English between 2002‐2012; (2) involved healthcare professionals; (3) were primary research. Commentary and anecdotal articles were excluded. The specific selection criteria were also applied, according to study design:

##### Quantitative studies

- Participants – healthcare professionals.
- Intervention – the introduction of mobile technologies.
- Outcomes – student, faculty, clinical staff or service and cost outcomes in education and/or clinical practice.
- Study design – comparison study, or a survey without a comparison group.

##### Qualitative studies

- Participants – healthcare professionals.
- Study design – Qualitative exploration of the perceptions or experience of students, faculty members or clinical staff about mobile technologies.

In this review, we categorized the educational outcomes according to Kirkpatrick's four‐level training evaluation model (Kirkpatrick 1994). When moving from level 1 to level 4, the methodologies required to achieve the outcomes tend to become more complex, however, the potential benefits and impacts for patients and healthcare organizations are greater. Four levels include:

- level 1 reaction: participants’ initial reactions or satisfaction, usually assessed through surveys and focus groups.
- level 2 learning: the amount of knowledge and skills that participants learnt, usually assessed through pre‐/post tests, observations and interviews.
- level 3 transfer: participants’ use of the knowledge and skills gained in everyday life, usually assessed through observations, interviews and surveys.
- level 4 dissemination and value to the organization: cost‐effectiveness and organisational benefits. Assessment for this level is not clearly defined but the more qualitative approaches using action research and critical incidents were seen to be a better approach to this level.

The focus of this evaluation model is on measuring four kinds of outcomes that should result from a highly effective training programme. There is a potential outcome line that ends with the level four results:

Training programme → Reactions → Learning → Transfer (behaviour) → Dissemination and value to the organization (increased productivity and profits).

Learning (level 2 outcomes) and transfer of learning (level 3 outcomes) are unlikely to occur unless participants have positive attitudes towards the training programme (level 1 outcomes). For dissemination and value to the organization (level 4 outcomes) to take place, there must be many intervening factors involved. This means that we should not be overly optimistic in expecting large level four outcomes from single training programmes.

### Search outcome

**Figure 1:**
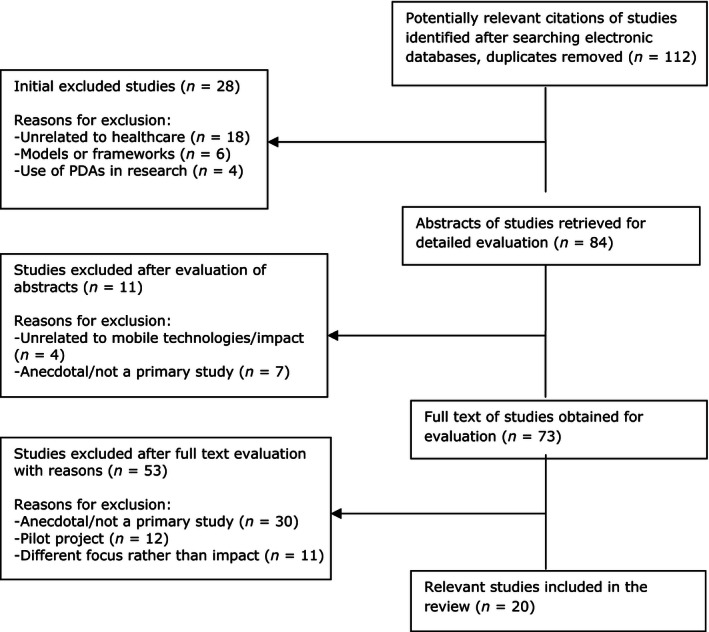
Flow chart of study selection process.

The titles and abstracts of 112 potentially relevant studies were independently reviewed by two reviewers (PG and KW) against the selection criteria and reasons for exclusion after evaluation of abstracts were recorded. Any discrepancies were discussed with a third reviewer (HW) to reach a final agreement. The full texts of 73 studies were retrieved and reviewed in further detail. Of these, 53 articles were excluded as they did not meet the inclusion criteria. Consequently, 20 primary studies published between 2002–2012 were identified and included in the review (Figure 1). These included: 17 quantitative (4 comparison studies; 13 descriptive surveys or studies without a comparison group) and three qualitative studies.

### Quality appraisal

Quantitative studies were critically evaluated based on a framework (Table 1) recommended by Thomas *et al*. (2003) while the consolidated criteria for reporting qualitative research (COREQ) (Table 2) was used to appraise the rigour of the qualitative studies (Tong *et al*. 2007). Thomas *et al*.'s framework was developed to be an appropriate quality assessment tool to encompass a variety of research designs (not only randomized controlled trials but also non‐randomized studies). It has been proved to have good content and construct validity and intrarater reliability and is relatively easy to use (Thomas *et al*. 2004). The quality of the studies varied.

**Table 1:** Checklist for assessing the quality of quantitative studies

Criteria	Strong	Moderate	Weak
1. Selection bias			
2. Study design			
3. Confounders			
4. Blinding			
5. Data collection methods			
6. Withdrawals and dropouts			
7. Intervention integrity			
8. Analyses			

**Table 2:** Consolidated criteria for reporting qualitative studies (COREQ)

Criteria	Yes	Partial	No
Domain 1: Research team and reflexivity Personal characteristics Relationship with participants			
Domain 2: Study design Theoretical framework Participant selection Setting Data collection			
Domain 3: Analysis and findings Data analysis Reporting			

#### Quantitative studies

Quality was relatively weak: three studies using a comparison group (Miller *et al*. 2005, Greenfield 2007, Flannigan & McAloon 2011) were non‐randomized quasi‐experimental studies. Only one study (Tews *et al*. 2011) applied the procedure of randomization but suffered with a small sample size of 22. Uncontrolled pre‐ and post tests designs were used in most studies. The included studies did not report details of confounding variables so there was insufficient information provided regarding whether or not groups were comparable at baseline. The areas such as selection bias, blinding and allocation concealment and data collection methods were poorly reported. In addition, all studies did not use strict ‘intention to treat’ analysis and did not explain how missing data and/or deviation from protocol or withdrawals/dropouts were analysed.

Thirteen surveys generally described clear objectives and design. However, sample sizes of these studies were often small (*n* = 10‐3306) and few studies attempted to explore the participants’ and non‐responders’ representativeness of the target population. Most studies involved non‐validated questionnaires or were unclear whether the measurement tools were valid or not. Therefore, it is recognized that the generalizability of these studies was limited.

#### Qualitative studies

All three studies (Garrett & Jackson 2006, Fisher & Koren 2007, Garrett & Klein 2008) had a clear description of aims and study design as well as an explicit sampling rationale. In these studies, the data collection and analysis methods were often clearly and transparently described. All studies obtained ethical approval but only one study (Garrett & Jackson 2006) explicitly reported possible ethical issues involved throughout the study. No studies included any consideration of reflexivity, however, most findings were presented clearly and the research provided valuable qualitative evidence.

### Data abstraction

A data abstraction form was developed using the Centre for Review and Dissemination (CRD) guidelines for undertaking reviews in health care (Centre for Reviews and Dissemination 2008). The form was used to record full study details and guide decision about the relevance of individual studies to the review questions. Similar information was abstracted for all studies and included: study design, setting, speciality, aim, study participants, results and conclusions. Data were abstracted and charted by PG and checked by KW or HW. Disagreements were addressed through consensus. Table 3 presents a full description of the included studies according to study design. Qualitative comments reported in quantitative studies were jointly extracted but we focused on the quantitative results.

**Table 3:** Overview of the included studies (*n *=* *20)

Reference/study design/setting	Speciality/aim	Study participants	Results Only the findings relating to impact/outcome are reported
Quantitative (*n *=* *17)			
Carroll and Christakis (2004) Survey USA	Paediatrics To determine the percentage of paediatricians using PDAs and computers, to determine perceived strengths and weaknesses of PDAs and to explore characteristics associated with beliefs and use	1185 paediatricians	35% of respondents currently use PDAs at work and 40% currently use PDAs for personal use. Of those using PDAs, the most commonly used apps were for drug reference (80%), personal scheduling (67%) and medical calculations (61%). Those using PDAs were more likely to believe that PDAs can decrease medical errors and increase efficiency.
De Groote and Doranski (2004) Survey USA	Health sciences To determine how PDAs are used on an academic health sciences campus to define the level of training and support the library can provide to the students and faculty	352 medical residents and health sciences faculty	Sixty‐one per cent of survey respondents used PDAs. The address book, date book and calculator were the most common uses reported for PDAs. Residents also reported a high use of drug databases on their PDAs. Most survey respondents indicated they would like to learn more about clinical resources for PDAs.
Flannigan and McAloon (2011) Comparison study UK	Paediatric emergency To compare the use of a drug calculator on a smartphone with use of the British National Formulary for Children (BNFC) for accuracy, speed and confidence of prescribing	28 doctors and seven medical students in a paediatric department of a District General Hospital	The drugs calculator on the smartphone was more accurate than the BNFC, with 28.6% of participants being able to correctly prescribe an inotropic infusion using the BNFC and 100% of participants being able to do so using the drugs calculator on the smartphone (*P *<* *0.001). The smartphone calculator was 376% quicker than the BNFC with the mean time saved being 5 min and 17 s per participant (*P *<* *0.001). Participants were more confident in their prescription when using the drugs calculator on the smartphone with a mean confidence score of 8.5/10 compared with 3.5/10 when using the BNFC (*P *<* *0.001).
Franko and Tirrell (2012) Survey USA	27 different specialties To evaluate the use of smartphones and smartphone apps	3306 providers (residents, fellows and physicians) at nation‐wide medical centres	Greater than 85% of respondents used a smartphone. Over half of the respondents reported using apps in their clinical practice; the most commonly used app types were drug guides (79%), medical calculators (18%), coding and billing apps (4%) and pregnancy wheels (4%). The most frequently requested app types were text/reference materials (55%), classification/treatment algorithms (46%) and general medical knowledge (43%).
George *et al*. (2010) Survey USA	Nursing To describe the use of PDAs by undergraduate and graduate nursing students during their educational process	48 nursing students in the undergraduate and graduate programmes	More than 79% of the participants used their PDAs at least weekly, with almost 50% using them daily. 96% of the participants reported using their PDAs in the clinical environment. 67% using PDAs in the classroom and 56% using PDAs for personal use. The drug guide was the most frequently used app by students (98%), followed closely by the medical dictionary (83%). 71% of the participants indicated that PDA use improve their efficiency. 77% thought that using PDAs as a student would contribute to their future use of handheld technology. 100% indicated that they found PDAs to be an effective educational tool.
Greenfield (2007) Non‐randomized quasi‐experimental study USA	Nursing To determine whether nursing medication errors could be reduced and nursing care provided more efficiently using PDA technology.	87 junior and senior undergraduate nursing students	PDA (experimental) group answered the six questions (three medication administration calculations and three clinical decisions based on medication administration) with greater accuracy and speed than did the textbook (control) group.
Kenny *et al*. (2009) Survey supported by interviews Canada	Nursing To evaluate the potential of mobile learning in nursing practice education	17 students in a nursing practice education course taught at the end of third year	Participants reported positively on the usability of the mobile devices, finding them easy to learn, readily portable and the screen size sufficient for mobile specific programmes. However, they had difficulty with the wireless connectivity and, despite an initial orientation, did not have time to fully learn the devices in the context of a busy course. It is not clear if students can effectively use the social technology provided by such devices or if mobile learning can support interaction between instructors and learners in this context.
Khan *et al*. (2007) Survey USA	Paediatric and emergency medicine To investigate the current PDA usage patterns of the residents and their interest in future PDA‐based applications	60 paediatrics and emergency medicine residents	82% of the PDA users reported using the device several times a day and 16% used them a few times a week. The most commonly used apps included the simple calculator (81%), drug references (80%), medical calculators (75%), electronic textbooks (66%) and schedule and contact information (42%). Residents showed interest in using PDA apps for procedure logs, patient tracking and prescription writing. No significant differences were noted in the frequency and expertise of using PDAs between the paediatric and emergency medicine residents (*P *=* *0.29).
Maag (2006) Survey USA	Nursing To explore students’ satisfaction with the academic podcasts as an emerging mobile learning tool	1st survey: 34 undergraduate and graduate nursing students 2nd: 33 undergraduate nursing students 3rd: 43 undergraduate and graduate nursing students	The students were generally satisfied with the availability and use of educational podcasts. Podcasts assisted their learning and provided valuable learning experiences.
Miller *et al*. (2005) Pre–post comparison study USA	Nursing To report on PDAs as a means to prepare nurse professionals who value and seek current information	58 second‐degree nursing student completing the pre‐intervention survey and 46 the postintervention survey	Results of this study support PDAs as an effective student learning resource, especially for reference materials. The student group with PDAs had increasing numbers of questions associated with clinical situations and a greater recognition of the need to use current resources. Students made substantial use of their PDAs and health team members, while decreasing reliance on textbooks and clinical faculty. Students’ use of and satisfaction with this technology is linked to access speed and readability.
Morris *et al*. (2007) Survey USA	Medicine To understand PDA usage and training in family medicine residency education	598 residents, fellows and full‐time physician faculty members	Use of PDAs is common among residents (94%) and faculty (79%). A total of 96% of faculty and residents report stable or increasing frequency of use over time. The common barriers relate to lack of time, knowledge and formal education. A total of 52% of PDA users have received some formal training. The majority of users report being self‐taught. Faculty and residents prefer either small‐group or one‐on‐one settings with hands‐on, self‐directed, interactive formats for PDA training.
Ranson *et al*. (2007) Case study USA	Primary care, nephrology, cardiology, emergency medicine, & endocrinology To describe use of (1) PDAs in patient care and (2) a PDA version of a learning portfolio in reflection on practice and medical education	10 practising physicians & specialists	All physicians accessed the system after training. Information accessed by PDA was used for clinical decisions support, patient education and teaching medical students. Use of the PDA version learning portfolio prompted physicians to reflect on changes in clinical practice.
Rothschild *et al*. (2002) Survey USA	Medicine To evaluate the clinical contribution of a palmtop drug reference guide – ePocrates Rx	946 randomly selected ePocrates Rx users	Physicians reported that ePocrates Rx saves time during information retrieval, is easily incorporated into their usual workflow and improves drug‐related decision making They felt that it reduced the rate of preventable adverse drug events.
Stroud *et al*. (2005) Survey USA	Nursing To describe the prevalence and patterns of use of PDAs by nurse practitioner (NP) students and faculty, examine relationships between patterns of use of PDAs and demographic characteristics of NP students and faculty and describe patterns of use of PDAs that support evidence‐based practice (clinical scholarship)	227 nurse practitioner students and faculty	A total of 67% of the participants (*N *=* *153) used PDAs. Use was higher among men (82%) than women (64%). On average, respondents who used a PDA had been using it just over a year (M = 13 months). Respondents reported using a PDA most days of the week (M = 5 days). The top three medical software programs identified by respondents as the most useful in clinical practice were ePocrates Rx (82%), Griffith's 5‐Minute Clinical Consult (26%) and MedCalc (22%). Use of the PDA clearly facilitated both student and faculty access to accurate and current knowledge. Most participants (96%) related that PDA use supported clinical decision making.
Stroud *et al*. (2009) Survey USA	Nursing To describe the prevalence and patterns of use of PDAs among active nurse practitioners	126 nurse practitioners	A total of 64% of participants used PDAs. A drug reference was reported to be the most useful and frequently installed application. A large majority of PDA users believed that PDA use supported clinical decision making (91%), promoted patient safety (89%) and increased productivity (75%). A total of 62% predicted that PDA use would change their practice within the next 5 years.
Tews *et al*. (2011) Comparison study USA	Emergency medicine To evaluate medical students’ case presentation performance and perception when using mobile learning technology in the emergency department	22 fourth‐year medical students randomized to receive or not to receive instruction by using the iPod Touch video	There was a statistically significant improvement in presentations, when the videos were viewed for the first time (*P *=* *0.032). There was no difference when the presentations were summed for the entire rotation (*P *=* *0.671). The reliable (alpha=0.97) survey indicated that the videos were a useful teaching tool and gave students more confidence in their presentations.
Walton *et al*. (2005) Survey UK	Community health To explore the potential for mobile technologies to give health students in the community access to learning resources	49 students on the health visiting/community nursing/school nursing course	Mobile technologies were mainly being used for clinical rather than learning applications. The students showed a low level of awareness of the various mobile technologies but placed great importance to accessing learning resources from the community. The most beneficial aspects of mobile technologies were seen as improved access to information, followed by improved contact with the university.
**Qualitative (** ***n *** **=** *** *** **3)**			
Fisher and Koren (2007) Focus group USA	Nursing To explore the perceptions of students lived experience using a PDA in clinical practice at the point of care in undergraduate nursing clinical education	28 third and fourth year of nursing undergraduate students in four focus groups	The integration of PDA technology into a clinical practicum was successful and positively viewed by the junior and senior students Six themes were identified: information resource; retaining information; clinical critical thinking; professional image; communication skills and quality of care.
Garrett and Jackson (2006) Qualitative evaluation Canada	Nursing and medicine To design, implement and evaluate a PDA‐based tool to support reflective learning in practice	Six final year nurse practitioner students and four final year medical students	The students on average used the apps for a total of 68 min per week. The PDAs were mainly used as electronic reference tools rather than data recording and communications devices. The use of PDAs was limited by the handwriting user interface. Although they acknowledged the value of professional reflection, the use of the guided reflection process was not regarded as a useful tool by the students. The value of the PDA to help prevent clinical isolation and support clinical learning was viewed positively.
Garrett and Klein (2008) Qualitative interpretivist Canada	Nursing To explore advanced practice nurses’ perceptions on the value of wireless PDA technologies to support their practice	43 nurse practitioners, clinical nurse specialists completing survey, two focus groups of 12 nurse practitioner students (24 total) and four information technology managers participating individual interviews	Wireless PDA's use supports the principles of pervasivity and is a technology rapidly being adopted by advanced practice nurses. Nurses identified improved client care as the major benefit of this technology in practice and the type and range of tools they identified included clinical reference tools such as drug and diagnostic/laboratory reference applications and wireless communications.

### Synthesis

The studies that met the selection criteria measured various outcomes ranging from clinical outcomes to educational outcomes. Therefore, it was decided not to pool the results using meta‐analysis but summarize them descriptively. Despite the current lack of guidance on the synthesis of diverse data sources in a sing review (Goldsmith *et al*. 2007), we decided to adopt a robust two step approach. The first step in the synthesis process was to construct a tabular summary of all studies related to key information including the study designs, countries, aims, specialist areas, study participants and results (Table 3). An overarching synthesis was subsequently carried out to bring quantitative and qualitative evidence together to explore the impact of mobile technologies further on both clinical outcomes (Table 4) and educational outcomes (Table 5).

**Table 4:** Impact of mobile technologies on clinical practice outcomes

Clinical outcomes	Evidence of impact identified in the qualitative studies	Evidence of impact identified in the quantitative/survey studies
Medical residents and physicians’ usage and perceived needs for PDAs		Increased trends of use of PDAs in clinical practice, decisions support, patient education, teaching medical students and increased interest for future apps (Franko & Tirrell 2012, Khan *et al*. 2007, Morris *et al*. 2007, Ranson *et al*. 2007, De Groote & Doranski 2004) Decreased medical errors and increased efficiency (Carroll & Christakis 2004)
Use of PDAs in nursing clinical education and practice	Improved professional image and quality of care (Fisher & Koren 2007) Improved client care and increased use of clinical reference tools (Garrett & Klein 2008)	Supported clinical decision making (Stroud *et al*. 2005) Improved access to information and improved contact with the university (Walton *et al*. 2005) Supported clinical decision making, promoted patient safety and increased productivity (Stroud *et al*. 2009)
Use of handheld devices in drug prescription		Increased accuracy, speed and confidence (Flannigan & McAloon 2011) Improved access to drug information, practice efficiency, drug‐related decision making and patient care (Rothschild *et al*. 2002)

**Table 5:** Impact of mobile technologies on educational outcomes

Educational outcomes	Evidence of impact identified in the qualitative studies	Evidence of impact identified in the quantitative/survey studies
Use and usefulness of mobile technologies to nursing students	Developed information resources, critical thinking and enhanced communication skills (Fisher & Koren 2007, level 1)	Improved efficiency (George *et al*. 2010, level 1) Increased accuracy and speed (Greenfield 2007, level 2) Enhanced learning experience and students’ satisfaction (Maag 2006, level 1; Miller *et al*. 2005, level 1; Kenny *et al*. 2009, level 1)
Use of a mobile clinical e‐portfolios by nursing and medical students	Improved clinical learning and engagement (Garrett & Jackson 2006, level 1)	
Use of handheld mobile technologies in medical education and training		Improved case presentation performance and confidence (Tews *et al*. 2011, level 2)

## Results

The studies (17 quantitative and three qualitative studies) had been conducted in a range of countries: 15 studies (14 quantitative and one qualitative) were set in USA; two quantitative studies were conducted in the UK; three studies in Canada (one quantitative and the other two qualitative). The review showed that use of mobile technologies has primarily been focused in the studies conducted in the USA. It is also apparent that in the healthcare context, the PDA is the most commonly used mobile technology up to the search date of this review.

The studies were undertaken in a variety of specialist areas, including paediatrics, emergency medicine, nephrology, cardiology, endocrinology and primary care. Nine studies evaluated the use and impact of mobile technologies among medical staff and medical students primarily at the undergraduate level and in the hospital environment, while eleven studies concentrated on nurses and pre‐registration nursing students. Other HCPs were less well represented. Only one study focused on the potential of mobile technologies to meet the needs of community health students on access to learning resource (Walton *et al*. 2005). The use of mobile technologies in emergency medicine was evaluated in three studies (Khan *et al*. 2007, Flannigan & McAloon 2011, Tews *et al*. 2011), of which two studies had a focus on paediatrics and emergency medicine (Khan *et al*. 2007, Flannigan & McAloon 2011).

There would appear to be equal importance of mobile technologies in both clinical applications and learning applications. Outcomes measured in the included studies varied but generally fell into the following two categories: clinical practice outcomes and educational outcomes. Table 4 presents the summary of impact of mobile technologies for clinical practice outcomes. Table 5 illustrates that mobile technologies have indicators of impact on educational outcomes.

In the clinical setting, several quantitative/survey studies identified that there were increasing trends for use of PDAs in clinical practice, decisions support (e.g. Rothschild *et al*. 2002, Stroud *et al*. 2005, 2009), patient education, teaching medical students (e.g. Khan *et al*. 2007, Morris *et al*. 2007, Franko & Tirrell 2012) and the development and use of future software applications. Four studies suggested that the use of PDAs had resulted in decreasing medical errors, increasing efficiency and promoting patient safety (Rothschild *et al*. 2002, Carroll & Christakis 2004, Stroud *et al*. 2009, Flannigan & McAloon 2011). Two qualitative studies suggested that PDAs had an impact on improved professional image and improved patients’ quality of care (Fisher & Koren 2007, Garrett & Klein 2008).

In terms of educational outcomes, the use of mobile technologies is concentrated on learning experience and students’ satisfaction (e.g. Miller *et al*. 2005, Maag 2006, Kenny *et al*. 2009). Improvements in learning were evident including learning accuracy and efficiency (Greenfield 2007, George *et al*. 2010), examining critical thinking and communication skills (Fisher & Koren 2007) and clinical learning and engagement (Garrett & Jackson 2006, Tews *et al*. 2011).

According to Kirkpatrick's four‐level training evaluation model, most of the studies included in the review measured level 1 – reaction outcomes which could be potentially biased by self‐perception. Only two quantitative studies assessed level 2 – learning outcomes (the amount of skills and knowledge that participants learnt) and indicated increased accuracy and speed (Greenfield 2007) and improved case presentation performance and confidence (Tews *et al*. 2011). No level 3 and 4 outcomes were measured in any included studies.

## Discussion

Twenty studies were identified and included in the review. However, methodological quality of these studies was relatively weak, especially in the studies with quantitative design. The findings of the review showed positive outcomes of using mobile technologies in both education and clinical settings. However, all studies were conducted in western, developed countries and were US centric. Limited evidence has been found on evaluation of their use and incorporation into healthcare professionals’ educational programmes, particularly allied health professionals’ education and training and its impact on patient outcomes and learning outcomes. These will be further discussed in the following three areas: the use of mobile technologies to aid engagement with learning; benefits of mobile technologies in clinical practice; adoption of mobile technologies in health care and barriers to mobile technology adoption.

### Use of mobile technologies to aid learning engagement

A growing body of literature draws attention to the potential of mobile technologies for the support of learning. A review of the impact of PDAs highlighted that the integration of PDA technology into medical education has a valuable contribution to residency training programmes, particularly graduate medical training (Tempelhof 2009).

Several articles also showed that integration of mobile technologies in nursing curricula allowed students to actively participate in different learning contexts and reinforce what they have learnt at any time or any location (Miller *et al*. 2005). Personal mobile devices such as smartphones can be used for immediate and constant access to information or materials, current evidence and guidelines in academic and clinical settings. An advantage was that students were able to view instructional videos before performing clinical tasks and timely approach their instructor via text message (Maag 2006, Kenny *et al*. 2009). A qualitative study in the review (Fisher & Koren 2007) conducted with 28 students in the third and fourth year of an undergraduate baccalaureate nursing clinical education programme found that the PDAs were successfully integrated into nursing education in the use of several reference resources such as drug guides/administration, medical dictionaries and patient information materials.

### Benefits of mobile technologies in clinical practice

Accurate patient care documentation and information are increasingly emphasized in health care. Frequently updated clinical guidelines further challenge healthcare professionals’ efficiency in daily practice. Sophisticated handheld devices have been developed and used to store patient information as well as monitor and keep healthcare professionals informed about the condition of their patients. A systematic review of surveys (Garritty & El Emam 2006) demonstrated that a PDA was more likely to be accepted and used among those physicians and residents who were younger and those who were working in large and hospital‐based practices. Although PDAs could not store or organize large graphics and patients’ entire medical records, they have played a significant role in managing certain amount of electronic documentation and accessing it at the point of care easily. However to inform the development and use of mobile technologies such as smartphones and tablets, more studies with high quality design investigating the effectiveness and efficiency of using mobile technologies for specific tasks are needed.

The literature suggests that use of mobile technologies saves clinicians time on information access and retrieval and allows them to spend more time in patient care (Rothschild *et al*. 2002, Flannigan & McAloon 2011). This mobile technology can help healthcare professionals to enhance patient care by improving information management, supporting evidence‐based decision making and accessing patient data remotely (Carroll & Christakis 2004, Garrett & Klein 2008, Stroud *et al*. 2009). A review (Lindquist *et al*. 2008) including 48 articles on the usage of PDAs among healthcare personnel and students showed that PDAs were used in patient care with varied frequency. The immediate access to drug and medical information potentially improves patient care. However, there is no robust evidence as most studies included in Lindquist *et al*.'s review are descriptive and only six randomized controlled trials. The review suggested that the PDA appeared to be a useful tool for health care personnel and students. There is a need for more intervention studies, action research and studies with different healthcare professionals to further identify the appropriate functions and applications of the PDA.

### Mobile technology adoption in health care and its barriers

Numerous studies have demonstrated cons iderable advantages of mobile technologies including wireless connectivity and therefore, a widespread adoption of this technology in health care (Franko & Tirrell 2012). A recent survey found a higher adoption rate of mobile technologies among physicians than general consumers. Lu *et al*. (2005) showed that more nurses in the USA and Canada were using PDAs than physicians in 2003. Previous reports proposed that over 70% of all medical residents was now operating a PDA for daily clinical support, with a 60% increase rate of use since 2001 (Barrett *et al*. 2004). In 2004, a survey of pharmacists found that 26% were using handheld computers on a daily basis, another example of early adopters in health care (Balen & Jewesson 2004).

Although there is evidence that mobile technologies in medicine, are used widely, several barriers to a more general adoption are evident which could discourage full use. These include personal factors (such as age, ability to comfortably use technology and devices and knowledge and skills), usability barriers (e.g. screen size), maintenance and security concerns, lack of technical support and insufficient training. It is recognized that many healthcare professionals need to be made aware of the variety of potential uses for mobile technologies. A scheduler alarm system can be set up to remind forthcoming appointments or meetings. Address books and to‐do lists can help organize and synchronize departmental tasks. Document readers are important software applications, which enable healthcare professionals to view any documents in text files such as medical references (George *et al*. 2010).

Concerns have been raised about patient confidentiality during use of handheld devices. Password is considered as the most commonly used approach to protecting patient data (Bogossian *et al*. 2009). Another important factor that may affect the adoption of mobile technologies into the medical setting is cost due to the expense of handheld units themselves, the software, the network and support. It may be assumed that cost will be returned through decreasing charting time, errors reduction and more time left for patient care (Eley *et al*. 2008). However, there is very limited evidence of cost‐effectiveness before and after adoption of mobile technologies. In addition, concerns about cross‐infection between patients could also be a barrier to uptake of a mobile device for intimate patient activities in healthcare setting, which has not been addressed in the studies included in this review (Brady *et al*. 2012, Mather *et al*. 2014, Trived *et al*. 2011).

Larkin reported that the physician was more likely to use the PDA if it could fit into his or her workflow seamlessly or if it did not require extra effort (Larkin 2001). Undoubtedly, advances in technology overcome some of the barriers to adoption such as advancements in memory storage, battery life, larger screens, wireless capabilities and high resolution displays. Other barriers may be eliminated by providing technical and financial support for the devices and software applications and increasing necessary training programmes for the clinicians (Dongsong & Adipat 2005).

### Methodological strength and limitations

The level of use of mobile technologies is expected to rise rapidly in healthcare providers’ practice. Currently, there has been limited evaluation of their use and incorporation into healthcare professionals’ educational programmes and its impact on learning outcomes. A comprehensive search strategy, rigorous selection criteria and systematic data extraction and critical quality assessment were applied to the whole review process. Since our search ended in 2012, the literature in this area has moved on. We believe, however, that our review, which rests on reproducible methods, provides a useful evidence base on the impact of mobile technologies in health practice and education. The review has demonstrated the important roles of mobile technologies in healthcare education and clinical practice settings and educators and healthcare professionals should be made aware of the potential benefits and the increasing trend of adopting mobile technologies to support and improve learning and clinical practice. Majority of the evidence reviewed is focusing on PDAs but not smartphones and tablet devices. The development of further devices opens the door to further implementation and evaluation.

This review has several limitations. Eligible studies might have been missed, although a thorough electronic and hand search was conducted. One possible explanation could be due to inconsistent terminology used in this field of research. There was also no attempt to search for unpublished studies and studies published in non‐English languages. In addition, studies which only focused on usage, usefulness, accessibility, acceptance of mobile technologies or the use of an element in PDA (such as an electronic barcode system in PDA and PDA‐based e‐portfolio) were excluded. It is acknowledged that the inclusion of those studies may provide further insight in the review.

Most studies in this review are descriptive, with weak study designs. Studies used a variety of outcome measures making it difficult to synthesize the findings. The fact that transfer of learning (level 3 outcomes) and dissemination and value to the organization (level 4 outcomes) were not measured in any included studies provided no evidence on the effect of these training programmes in behavioural changes and increased productivity and profit. The other major limitation is that the included studies relied on self‐reporting of learning rather than using assessment marks.

## Conclusion

The synthesis of the evidence on the impact of mobile technologies on education and clinical practice outcomes remains inconclusive due to the descriptive nature of the body of research available to date on this topic. The development of mobile technologies for healthcare professionals is expanding rapidly and benefits of mobile technologies in the education and practice of healthcare professionals have been articulated in the literature. This review suggested that mobile technologies in healthcare potentially improve access to information, enhance productivity and quality of care, reduce medical errors, increase engagement with learning in different contexts and promote evidence‐based decision making at the point of care.

## Recommendations for health care and research

These considerable benefits made a significant contribution to the increased trend in healthcare professionals’ adoption of mobile technologies. Although the increasing implementation of this technology appears impressive, limited evidence about the effect of mobile technologies on patient outcomes was identified through a comprehensive literature search. The topic is still under development and is in need of further research with robust design to evaluate the effectiveness and/or cost‐effectiveness of mobile technologies for enhancing care efficiency and patient outcomes and to explore expanding roles of mobile technologies and experiences of using new mobile technologies in improving healthcare education and practice among healthcare professionals and healthcare students.

## Conflict of interest

No conflict of interest has been declared by the authors.

## Author contributions

HW and KW were responsible for the study conception and design. PG performed a comprehensive literature search, study selection and review. HW and KW provided insightful guidance and double‐check in identification, collection and analysis of papers throughout this review. PG drafted the manuscript. HW and KW made critical revisions to the manuscript for important intellectual content. All authors approved the final manuscript and act as guarantors for the study.

All authors have agreed on the final version and meet at least one of the following criteria [recommended by the ICMJE (http://www.icmje.org/recommendations/)]:

- substantial contributions to conception and design, acquisition of data, or analysis and interpretation of data;
- drafting the article or revising it critically for important intellectual content.
